# Exosomes-based therapy of stroke, an emerging approach toward recovery

**DOI:** 10.1186/s12964-022-00919-y

**Published:** 2022-07-22

**Authors:** Fatemehsadat Seyedaghamiri, Leila Salimi, Dara Ghaznavi, Emel Sokullu, Reza Rahbarghazi

**Affiliations:** 1 Student Research Committee, Tabriz University of Medical Sciences, Tabriz, Iran; 2 Neurosciences Research Center (NSRC), Tabriz University of Medical Sciences, Tabriz, Iran; 3 Department of Neurosciences, Faculty of Advanced Medical Sciences, Tabriz University of Medical Sciences, Tabriz, Iran; 4 Stem Cell Research Center, Tabriz University of Medical Sciences, Imam Reza St., Golgasht St, Tabriz, Iran; 5 Department of Periodontics, School of Dentistry, Zanjan University of Medical Sciences, Zanjan, Iran; 6 Koç University Research Center for Translational Medicine (KUTTAM), Rumeli Feneri, 34450 Sariyer, Istanbul, Turkey; 7 Department of Applied Cell Sciences, Faculty of Advanced Medical Sciences, Tabriz University of Medical Sciences, Tabriz, Iran

**Keywords:** Stroke, Exosomes, Regeneration, Therapeutic modalities

## Abstract

Based on clinical observations, stroke is touted as one of the specific pathological conditions, affecting an individual’s life worldwide. So far, no effective treatment has been introduced to deal with stroke post-complications. Production and release of several neurotrophic factors by different cells exert positive effects on ischemic areas following stroke. As a correlate, basic and clinical studies have focused on the development and discovery of de novo modalities to introduce these factors timely and in appropriate doses into the affected areas. Exosomes (Exo) are non-sized vesicles released from many cells during pathological and physiological conditions and participate in intercellular communication. These particles transfer several arrays of signaling molecules, like several neurotrophic factors into the acceptor cells and induce specific signaling cascades in the favor of cell bioactivity. This review aimed to highlight the emerging role of exosomes as a therapeutic approach in the regeneration of ischemic areas.

**Video Abstract**

**Supplementary Information:**

The online version contains supplementary material available at 10.1186/s12964-022-00919-y.

## Background

Stroke is the second leading cause of human mortality death and the third reason of disability worldwide [1]. As a common belief, stroke is divided into ischemic and hemorrhagic types [2]. Ischemic stroke is more common assigning 80% of stroke cases [3]. Following a cerebral stroke, several cognitive and motor deficits occur depending on the severity and location of the lesion [4]. To be specific, relative and/or complete occlusion of the blood supply into the brain parenchyma can be occurred due to thrombosis or the rupture of cerebral arteries, leading to neurological symptoms [5]. Following the occurrence of stroke, several mechanisms like excitotoxicity, inflammation, loss of mitochondrial function, free radicals generation, and accumulation of misfolded proteins lead to neuronal death [6]. Besides, the untamed release of glutamate and over-activation of NMDA and AMPA receptors can exacerbate the conditions. A further influx of calcium into the cytosol can abrogate the cellular hemostasis, resulting in oxidative and nitrosative stress [7]. It was suggested that continuous and prolonged blood deprivation can lead to irreversible neuronal death located inside the ischemic zone. By contrast, neurons in the vicinity of the ischemic zone, known also as the penumbra, have a chance to restore their function and activity when the blood flow is reestablished [8].

Current ischemic stroke therapies are based on quick clot removal in affected vessels using tPA. Unfortunately, there are some limitations in cases that received tPA. For instance, tPA possesses a narrow therapeutic window and can contribute to massive hemorrhage in some patients about 4 to 5 h after administration [9]. Along with a therapeutic strategy, endovascular thrombectomy can reduce the mortality rate in stroke patients during the first 12 h via re-canalizing obstructed cerebral arteries [10]. The restoration of cerebral blood flow after a specific period may postpone regeneration capacity and impose brain repair implausible because of irreversible injuries related to reperfusion mechanisms. As commensurate with these descriptions, rapid restoration of tissue perfusion in stroke patients can improve outcomes in survivors [11].

In recent years, whole cell-based therapies have been introduced as a promising therapeutic approach for stroke recovery [12, 13]. Among different cell types, stem cells facilitate the improvement of neurological function in stroke survivors via differentiation capacity, juxtacrine and paracrine activities [14–16]. Despite these advantages, stem cell therapy has some limitations with potential risks. It has been shown that direct cell injection through a systemic pathway can increase the possibility of an intravascular clot [17]. Meanwhile, there are some reports demonstrating tumorigenicity at the site of injection using stem cells. Another problem related to systemic injection is the low capacity of transplanted stem cells to cross the BBB from the blood side to the brain parenchyma [18]. Considering these limitations, researchers and clinicians are searching for alternative approaches [19–21]. Stem cells and other mature cell types can shed a large volume of extracellular vesicles (EVs) namely Exo into the ECM, participating in paracrine cell-to-cell interaction. Due to unique physicochemical properties and diverse biological activities, such as anti-inflammatory and anti-apoptotic effects, Exo can recapitulate the regenerative capacity of stem cells and other supporting cells. Besides, it seems that Exo can be efficiently distributed inside the body because of their nano-sized features. Some authorities have claimed that Exo can be used as a natural biomarker to predict the severity of pathological conditions after injuries such as neurological disease [22].

## Biogenesis of Exo

In the scientific literature, EVs are classified into three type such microvesicles, exosomes, and apoptotic bodies. Based on the size, Exo are in the range of 30–150 nm and actively secreted from each cell. Among Exo, two different populations (small and large nanometer) are detectable which are 60–80 nm and 90–120 nm, respectively. Of note, a group of Exo with dimensions of less than 35 nm is called exomeres [23, 24]. From morphological aspects, Exo display a round or cup shape structure and harbors proteins, lipids, and nucleic acids. Inside the cell, Exo are released by the direct fusion of late endosomes/MVB with the plasma membrane [25]. As above-mentioned, like several tissues, Exo partake in the regulation of nervous system homeostasis. Other activities such as regulation of immune system response, angiogenesis, neurogenesis, myelination, and synaptogenesis [25, 26]. Based on molecular investigations, Exo can express specific surface markers such as several Tetraspanins, ESCRT proteins, HSPs, etc. [27]. Like different stem cell types, Exo are eligible to promote healing procedures inside the nervous system after ischemic changes via several mechanism transfers of neurotrophic factors, induction of angiogenesis, and neurogenesis in which the combination of whole cell-based transplantation with Exo exhibit significant regenerative capacity. Besides these effects, Exo can control the production of pro-inflammatory cytokines, gliosis, and astrocyte and microglia polarization in the injured sites [28, 29]. The existence of specific genetic cargo such as miRNAs inside Exo lumen helps these nanoparticles to exert efficient therapeutic effects upon reaching to the target cells [30, 31]. The composition of the exosomal miRNAs is different from the parent cell's miRNAs, meaning that specific miRNAs have evolved to pack in exosomes for performing their biological functions in the recipient cells [32]. Engineered Exo using genetic modalities can introduce particular miRNAs to target sites with specific restorative and protective pathways for facilitating post-stroke repair processes [29, 33, 34]. Despite advantages, it has been shown that there are some obstacles related to Exo therapy in in vivo conditions [35]. The rate of Exo transfer through the BBB is to assess after systemic injection. Although Exo can provoke immune response in less extent however the possibility of allogeneic and xenogeneic response is not unlikely. Some authorities have declared the occurrence of clot formation (thrombosis) and iatrogenic or insidious infections [36]. Besides, the possibility of latent viral infections should not be neglected during the preparation of Exo for therapeutic purposes [37]. Compared to artificial nanoparticles, Exo possess a large number of adhesion molecules that facilitate the adhesion, tethering, and transfer of Exo through the BBB [38]. Another problematic issue regarding the application of Exo in in vivo condition is associated with the heterogeneity of exosomal population that differs in size and cargo type [39].

**Fig. 1:**
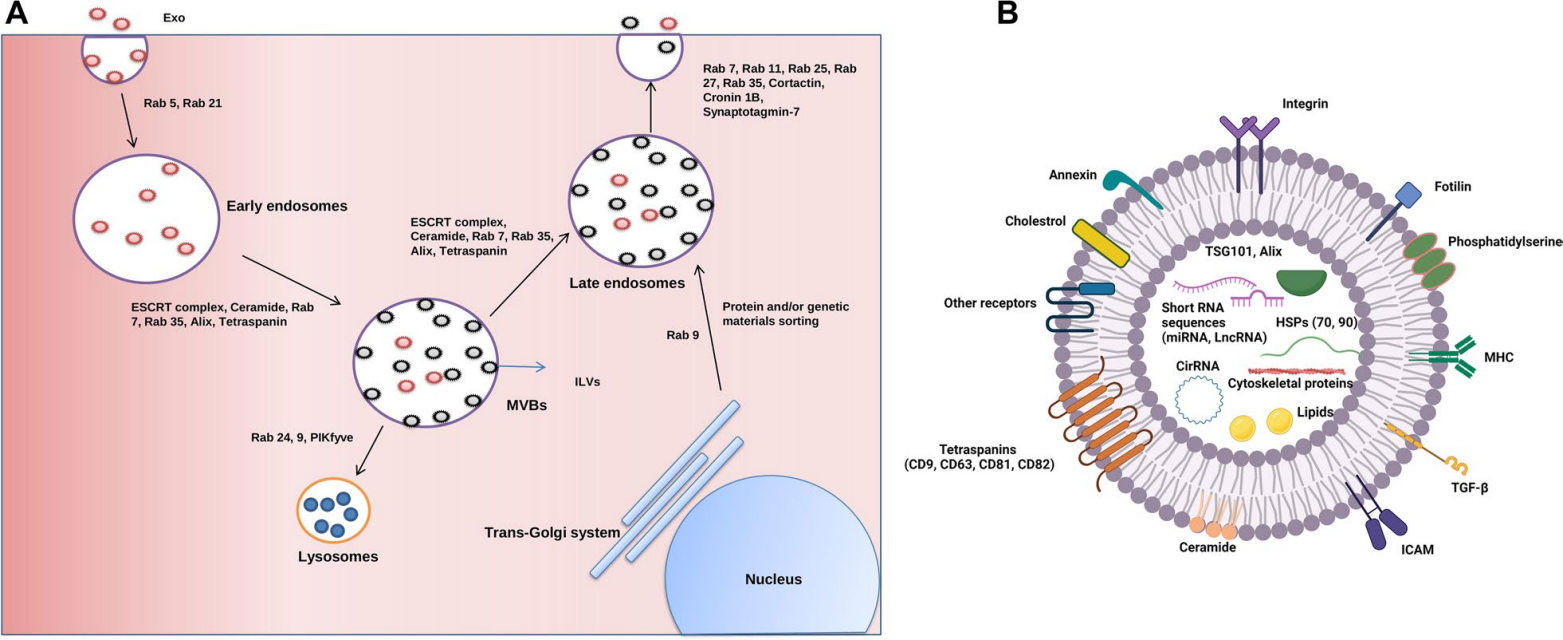
Intracellular machinery component of exosome (Exo) (**A**). biogenesis. Exo are generated inside endosomal vesicles. The maturation of early endosomes into multivesicular bodies (MVBs) leads to the formation of numerous intraluminal vesicles (ILVs). In the next steps, MVBs can be directed to lysosomal digestion and/or transformed into later endosomes which can fuse with the cell membrane. Upon release of ILVs into ECM, they are named Exo. Exo structure and cargo (**B**). Exo possess a Lipid bilayer with several ligands and receptors. Exo can transfer several signaling molecules such as lipids, proteins, and enzymes

Exo are classified as nano-sized EVs with lipid bilayer secreted into ECM in response to physiological and pathological conditions [40]. From molecular aspects, Exo are originated from endosomes and MVBs inside the cytosol [41] (Fig. 1). In the latter steps, these endosomes can fuse with lysosomes to degrade the exosomal cargo and/or fuse directly with the plasma membrane to release Exo into the ECM [42]. Molecular investigations have revealed that early endosomes can mature into late endosomes. Ultrastructural studies have shown that the invagination of the membrane in early endosomes and MVBs can form ILV which are known as Exo after releasing into the ECM [43]. Cell biological studies have indicated the participation of several types molecular of machinery in the process of Exo production inside each cell. For instance, ESCRT consisted of four different types of proteins including ESCRT-0, -I, -II, -III, plus the associated AAA-ATPase VPS4 complex can promote the formation and production of Exo [25]. Of note, ESCRTs -0, -I, and -II form heterogeneous oligomers with the ability to detect phospholipid and phosphatidylinositol 3-phosphate (PtdIns-3) P)-rich ubiquitin's on the surface of endosomes [44]. The existence of a specific subunit in the structure of ESCRT‐0 namely HRS can act as an adapter protein between the ubiquitylated cargo and ESCRT‐I [45]. The collaboration of ESCRT-I and ESCRT-II can drive membrane budding [45]. The assembly of ESCRT‐I and ESCRT‐II is located at the bud neck and recruits ESCRT-III [45, 46]. It is noteworthy to mention that ESCRT-III is not eligible to attach directly to ubiquitin. However, the presence of Bro1/Alix (ALG2-interacting protein X) can facilitate the binding of ESCRT-III to ubiquitin. It was suggested that attachment of ESCRT-III accelerates the invagination of ILVs into the MVBs lumen [44]. It seems that there are regulatory mechanisms that confine the activity of the ESCRT complex. For example, the addition of ESCRT‐III to ESCRT-I and II complex per se activates the AAA‐ATPase VPS4, leading to the disassembling of the ESCRT complex (Fig. 1) [45]. Along with ESCRT complex machinery, a ceramide-dependent pathway has been discovered in the context of Exo biogenesis. The activation of the ceramide-dependent pathway causes the conversion of sphingomyelin into ceramide by engaging sphingomyelinase. In line with the activity of the ESCRT complex, the recruitment of the ceramide-dependent axis help to fuse microdomains and promote ILVs formation inside the MVBs [43]. The exposure of each cell to several pathological and physiological conditions can affect the affinity and activity of exosomal machinery. For instance, continuous exposure of the cell to stress such as starvation and hypoxic condition can affect the number and intensity of MVBs, which eventually can lead to enhanced Exo secretion capacity [25]. It is thought that an increased Exo production during pathological conditions is a compensatory response to exclude the injured contents or to promote paracrine interaction with other cells. As abovementioned, the ESCRT complex actively partakes in the generation of ILVs inside the endosomes and MVBs. The intracellular trafficking of vesicular bodies is done by the activation of tetraspanins and a variety of cellular components, including primary MHC-II molecules [47]. The promotion of several Tetraspanin subsets in the format of micro-domains can sort selective cargo into exosomal lumen and exchange genetic and protein materials between cells [48]. Soon after Exo secretion, these nano-sized particles can be adsorbed by acceptor cells via different mechanisms. The existence of several signaling molecules on the surface of Exo (ligands) can activate a specific signaling pathway in recipient cells via specific receptors [43]. Physiochemical fusion of Exo with plasma membrane can also accelerate the discharge of Exo content into the cytosol or the fusion of Exo can promote the formation of early endosomes inside the recipient cells [49]. Upon the entrance of Exo into the acceptor cells and formation of endosomes, these structures can direct toward lysosomes and undergo digestion. In an alternative pathway, endosomes can mature to late endosomes can orient toward ECM [49, 50].

## Role of exosomes in neuro-inflammation post-stroke

**Fig. 2:**
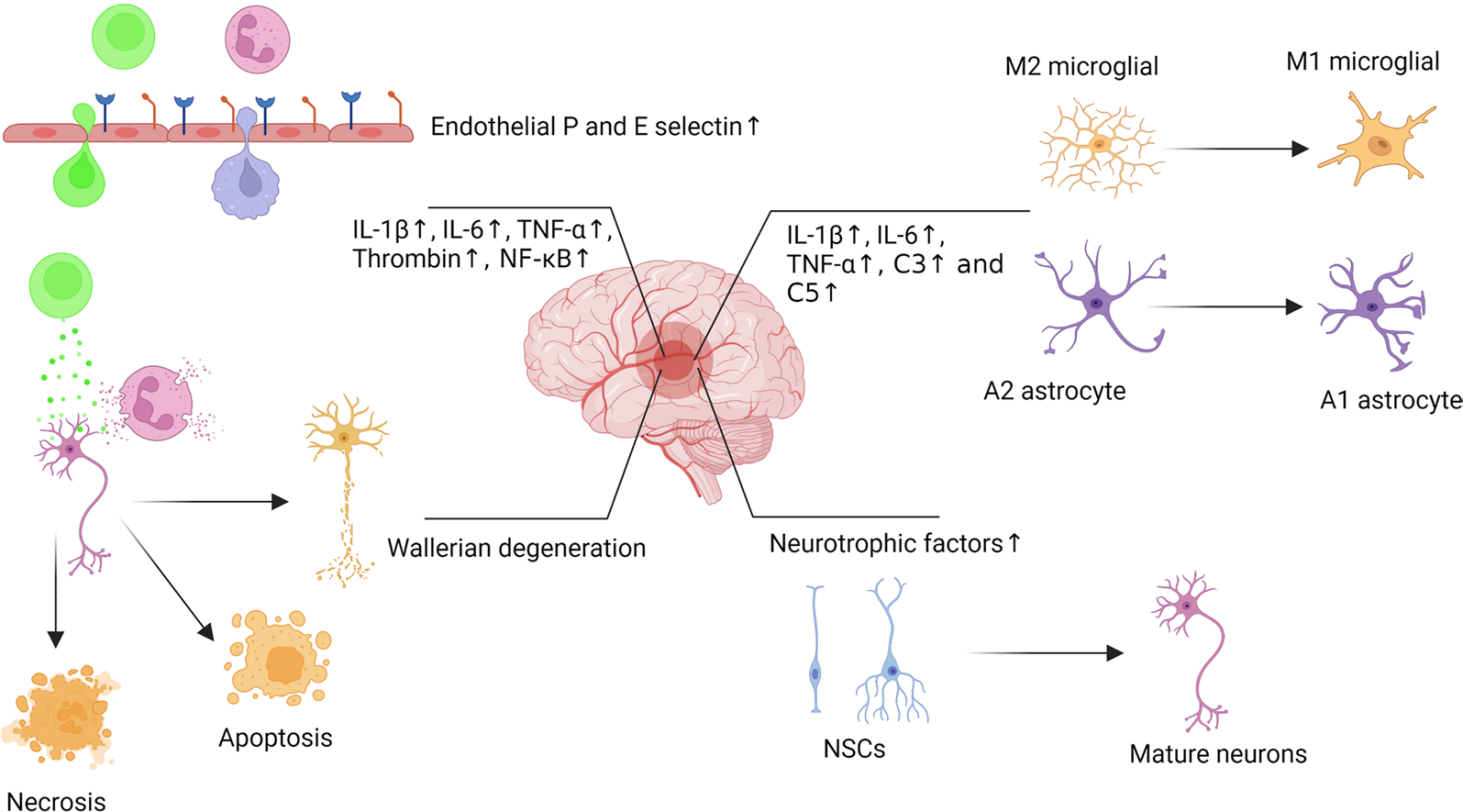
Neuroinflammation after brain stroke. Under ischemic conditions, glial cells can acquire a pro-inflammatory phenotype and release pro-inflammatory factors leading to neurological defects. Also, the activation of leukocytes leads to the release of pro-inflammatory factors, and injury of endothelium within the BBB, resulting in the propagation of inflammation into CNS parenchyma. The induction of adhesion molecules like P- and E-selectins can recruit and attach tightly leukocytes to the endothelial layer. These features can promote the formation of perivascular cuffing that can exacerbate inflammatory symptoms and increase stroke damage

**Fig. 3:**
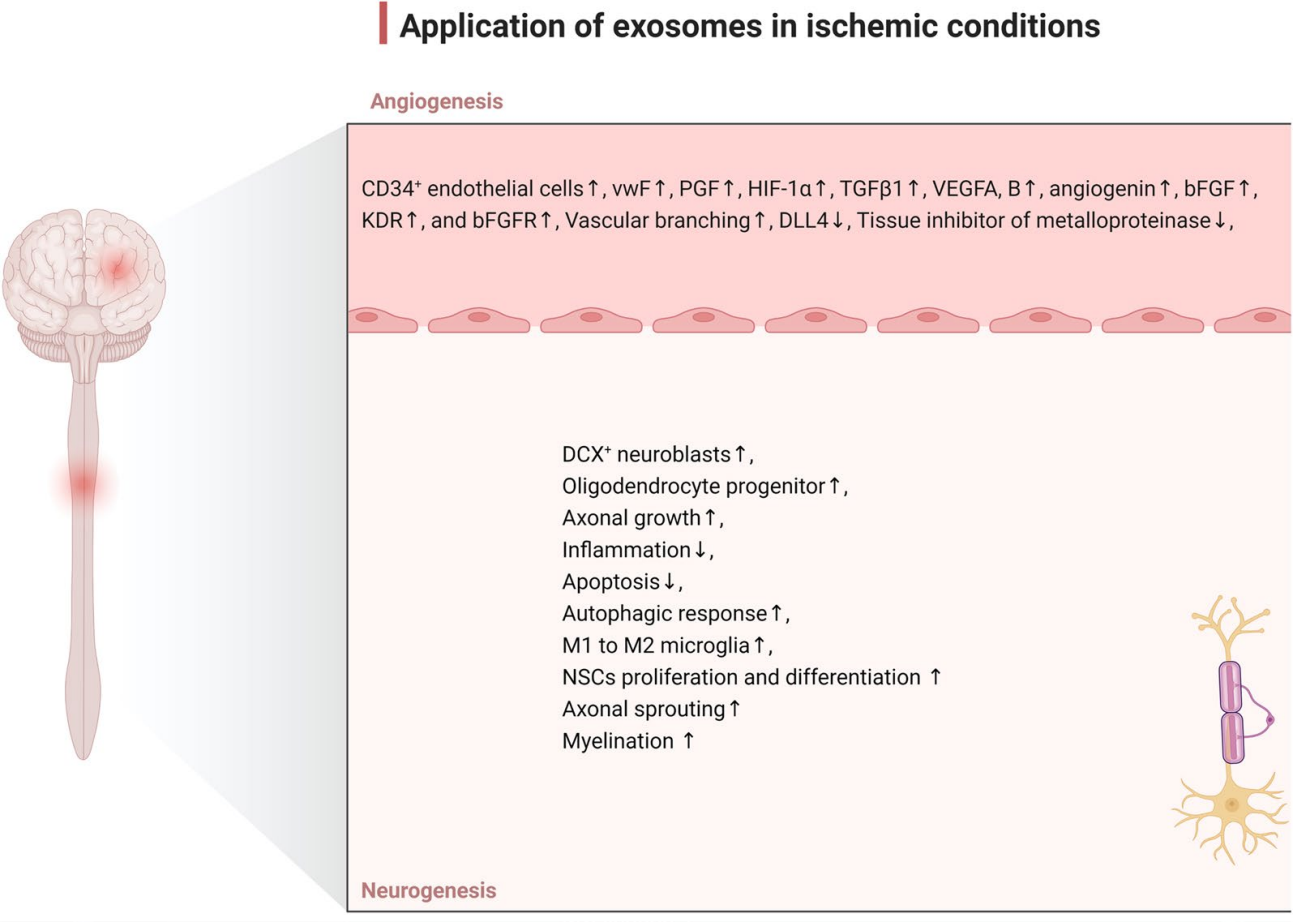
Several therapeutic outcomes after application of Exo in ischemic conditions such as stroke. Exo can promote angiogenesis via the regulation of several signaling pathways, leading to improved vascularization into the ischemic areas. Besides the angiogenic potential of Exo, these nanoparticles can induce neurogenesis via the proliferation and migration of NSCs. The proliferation of oligodendrocytes and the synthesis of myelin can help the injured neurons to overcome the insulting conditions. Due to the immunomodulatory properties, Exo promote M1 to M2 phenotype switching in microglia and reduce the production of pro-inflammatory cytokines

The promotion of neuro-inflammation following ischemic changes can exacerbate tissue injury and delay healing procedures [7]. Upon the occurrence of stroke, microglial cells can acquire an M1 type phenotype and release arrays of pro-inflammatory factors including IL-1β, -6, and TNF-α leading to the suppression of neurogenesis, neuronal apoptosis, and bulk neurological defects (Figs. 2, 3) [51]. Besides, the activation and recruitment of leukocytes and releasing of the pro-inflammatory factors from the damaged endothelium within the BBB leads to the propagation of inflammation into CNS parenchyma [7]. In such circumstances, the immune system cells are active for several days or weeks and therefore several inflammatory cascades are provoked. For instance, thrombin, a chemotactic factor, is released by neutrophils and monocytes which can stimulate NF-κB and the expression of cell adhesion molecules like P- and E-selectins (Figs. 2, 3). Moderate to the prominent expression of cell adhesion molecules can recruit leukocytes to tightly adhere to the endothelial layer and eventually form peri-vascular cuffing. Continuous accumulation of inflammatory cells at the periphery of blood vessels can exacerbate inflammatory symptoms and increase stroke damage [52–55]. Local increase of thrombin activates the complement system and via the stimulation of C3 and C5, inhibition of thrombomodulin, an anti-inflammatory factor, thereby enhancing neuro-inflammation. It is noteworthy to mention that thrombin can also aberrantly remodel the BBB structure and increase vascular permeability [56–59].

Along with these changes, there is evidence that peripheral inflammation induces exosomal release from the choroid plexus epithelium into the blood-cerebrospinal fluid (CSF). Soon after entry into the CSF, these Exo can ignite a neuro-inflammatory response via transporting their pro-inflammatory cargo into the astrocytes and microglial cells [60]. As a correlate, this phenomenon is the reason for the participation of endogenous Exo in inflammation following stroke. Monitoring stroke patients' blood samples in the acute stroke phase shows elevation of enriched C-reactive proteins Exo presence. These Exo can induce the expression of certain cytokines and chemokines in macrophages [61]. It seems that the origin and type of cell can pre-determine the pro- and anti-inflammatory capacity of Exo. For example, MSC-derived Exo can attenuate inflammatory response following brain injury by suppressing IL-1β levels [62], leading to the inhibition of microgliosis and astrogliosis, and immuno-modulation [63, 64]. One reason would be that MSC-derived Exo enhance phenotype shifting in microglia from M1 to M2 by regulating the CysLT2R (an inflammatory receptor)-ERK1/2 pathway [51, 65]. There is a direct association between activation of the ERK1/2 pathway and the increase of inflammatory reactions by microglial cells. ERK1/2 pathway is responsible for iNOS activation induced by LPS or IFN-γ. Furthermore, inhibition of the ERK1/2 pathway could be attenuated inflammatory response by altering microglial M1/M2 polarization [66]. Subsequently, the microglial M2 phenotype improves neurological outcomes by diminishing inflammation, reducing brain infarct volume by releasing anti-inflammatory factors such as IL-4 and IL-10, increasing neuronal repair, and enhancing regeneration [51]. Interestingly, M2-type microglia Exo can prevent neuronal apoptosis (Fig. 2) [67]. These Exo can harbor high levels of miRNAs to modulate inflammatory responses after ischemia. Microglial derived Exo contain a large amount of miRNA-126-5p, miRNA-21-3p, and miR-212 which are involved in M1-to-M2 microglia polarization[51]. Cerebral thrombosis activates inflammation through thrombin-activated platelet-derived Exo and miRNA-223 followed by NF-κB and MAPK signaling pathway activation. Also, the miR-223 expression inside Exo in ischemic conditions inhibits phosphorylation of p38, JNK, and ERK, and suppresses NF-κB p65 transfer to the nucleus leading to down-regulation of adhesion molecules including ICAM-1 [68]. Thereby, adhesion molecules in clot formation inside blood vessels exacerbate inflammatory conditions [52–55]. So, inhibiting adhesion molecules generating Exo would have a promising effect on improving inflammatory damage during stroke.

## Exo and apoptotic neuronal cell death

The apoptotic cell death is responsible for a major ischemic brain injury. As a correlate, the inhibition of apoptotic cascade has therapeutic potential for neuroprotection after stroke [69]. The process of apoptosis after stroke is initiated with an increase in free radicals, ionized Ca^2+^ content of cytosol, and subsequent excitotoxicity. Depending on the type, age, and anatomical origin of brain cells, these events can cause different cell death types such as necrosis or apoptosis in injured neurons. As a common belief, the promotion of apoptotic signaling can lead to morphological and ultrastructural changes, cellular blebbing, and expression of death receptors which are recognized via phagocytic cells [70]. It is believed that MSC Exo can alleviate ischemia-associate injury via suppressing apoptosis, enhancing angiogenesis, and immune response regulation (Fig. 3) [71]. MSC Exo loaded with MiR-133b can exert bulk anti-apoptotic effects in the ICH rat model through ERK1/2, CREB, and RhoA modulation [72]. Under these conditions, that increases the survival rate of glomerular epithelial cells [73] and is considered a neuroprotective pathway, in ischemia–reperfusion injury [74]. Similarly, Xiao and co-workers demonstrated that endothelial cell Exo directly protects SH-SY5Y neuroblastoma against I/R injury by promoting cell growth, and migration, inhibiting cell apoptosis and promoting cell cycle and proliferation [75]. In a study, it has been shown that the load of miR‐134 on MSC Exo can suppress extrinsic apoptosis singling cascade via the regulation of Caspase 8, resulting in reduced oligodendrocyte apoptosis after OGD [76]. However, miR‐134 inhibitors increase the expression of procaspase‐8 and caspase‐8 cleaved product proteins induced by OGD [76]. Also, xenogenic adipose-derived MSCs Exo, decrease the expression of cleaved caspase 3 and cleaved PARP, (two indexes for apoptosis), γ-H2AX (an index for DNA injury), and cytosolic cytochrome C (an index for mitochondrial injury) in the lesion area, diminish brain infarct volume and have a neuroprotective effect after acute ischemic stroke [77].

## A critical role of exosomes and autophagy after stroke

Autophagy is a catabolic pathway that is performed to maintain cellular homeostasis and maintain the function of cells under normal conditions. Following the autophagic response, damaged metabolites and organelles are subjected to degradation via lysosomal activity and released into the ECM [78]. To this end, basal activity of autophagy is vital to cell function maintenance [78], and resistance in response to the pro-inflammatory niche [79]. To be specific, nutrient deprivation, hypoxia, ROS accumulation, and karyolysis can lead to an autophagic response in most cells after a stroke within the brain parenchyma [78, 80, 81]. The frustrated autophagic response can be raised even after brain ischemia/reperfusion insult and neuronal injury [82–84]. It is believed that autophagy is an early-stage player in the pathophysiology of stroke and inflammatory diseases via the regulation of immune cell homeostasis [80]. The production of some inflammatory biomolecules like TNF-α, IL-1α, and β can affect the progress of autophagy and vice versa [85]. In line with this statement, the inhibition of autophagy notably diminished the OGD-induced inflammatory response in in vitro milieu. Similarly, in vivo studies have demonstrated a significant decrease in the cerebral injury area of infarction via the acceleration autophagic response in the target sites [86]. The phenomenon of autophagy can be tightly regulated via several genetic materials such as miRNAs. For instance, miRNA-30d-5p expression is associated with autophagy inhibition. Because Exo can carry different miRNAs, it is logical to hypothesize that Exo are integral to the regulation of autophagy response. In this regard, exosomal miRNA-30d-5p associated with adipose-derived stem cells (ADSCs) successfully reversed OGD-induced autophagy injury under ischemic conditions by the suppression of Beclin-1 and Atg5, and acceleration of microglial polarization from M1 toward M2 type [86]. As such, transplantation of astrocyte-derived Exo increased neuronal survival rate; diminished OGD-induced apoptosis, and alleviated OGD-induced expressions of Caspase-3, Bax, and other inflammatory factors levels in hippocampal neuronal cell line HT-22 after injection into in C57BL/6 mice models of MCAO. Likewise, astrocyte Exo can similarly inhibit the apoptosis rate in neurons exposed to OGD stress via regulating autophagy [87]. From a molecular viewpoint, the transfer of exosomal miR-190b results in the control of the 3′-UTR region of the neurotropic gene autophagy-related gene 7 (Atg7) and the inhibition of OGD-induced autophagy [88]. Application of circRNA microarray analysis indicated astrocyte Exo can harbor a high-level expression of circRNA transcribed from the SHOC2 gene, named circSHOC2 (circ_0092670) preconditioned by ischemia. Of note, circSHOC2 acts as a sponge for miR-7670-3p, and up-regulates the transcription of SIRT1, leading to the suppression of gliomas apoptosis, modulation of neuronal autophagy, and ischemic brain injury [89]. Interestingly, an experiment has demonstrated that PEDF-loaded Exo derived from ADMSCs exerted therapeutic effects on cerebral I/R injury. Physiologically, PEDF is a neuroprotective protein with anti-inflammatory and antioxidant properties. Decoration of ADMSC Exo with this factor ceases apoptosis by suppression of Caspase-9 and -3 in OGD-exposed neurons. Increasing PEDF content in the Exo lumen can exert a protective effect via autophagic response under OGD condition [90]. Commensurate with these effects, exosomal modulation of astrocytes can be as an alternative modality in the alleviation ischemic changes.

## Role of exosomes on neurovascular remodeling after stroke

As previously documented, Exo exhibit a fundamental role in neurovascular remodeling following several pathologies [26]. Within the ischemic brain, the administration of MSC-derived Exo increases the migration of DCX^+^ neuroblasts from the SVZ to the ischemic penumbra. Besides, these Exo can enhance axonal density, and angiogenesis and increase the number of progenitor oligodendrocytes and mature oligodendrocytes, leading to myelination of injured axons [26, 91]. Under these conditions, the expression of vWF (endothelial cell marker), promotes local blood perfusion into the ischemic area and enhances neurite remodeling, and neurogenesis [29]. Some documents point to the fact that MSC Exo can directly affect axonal growth in cortical neurons by inhibiting Argonaut 2. It is believed that the existence of miR-17-92 can also result in axonal growth via the activation of the PTEN/mTOR signaling pathway [92]. Along with these statements, microRNA 133b-overexpressing MSCs produce Exo with the ability to improve neural plasticity, increased functional recovery and neurite remodeling/brain plasticity, enhanced neurite branching and elongation of cultured cortical embryonic rat neurons in the ischemic boundary area with a contribution from a stimulated secondary release of neurite-promoting exosomes from astrocytes [31].

Inadequate blood supply during stroke causes neuronal death and serious brain damage in the lesion area. The activation of angiogenesis is a process by which new blood vessels are formed as a compensatory mechanism to cope with this complication and this process is vital for the repair of the ischemic lesion. Therefore, activation of angiogenesis is an effective therapeutic strategy to ameliorate the destructive effects of stroke [93–95]. Angiogenesis therapy is used to treat ischemic diseases such as ischemic stroke. Therapies used for angiogenesis therapy include protein/gene, stem/progenitor cell, and exosome/microvesicle therapies [95]. Exo have been introduced in recent years as new agents for enhancing angiogenesis [96]. These particles transport several proteins and genes with pro-angiogenic properties to areas affected by ischemia. Interestingly, it has been shown that Exo from different cell sources such as MSCs, cardiac progenitor cells, endometriotic stromal cells, and human-induced pluripotent stem cells (iPSCs) can boost angiogenesis [95, 97]. Have been proven that MSCs Exo contain growth factors, cytokines, and cell adhesion molecules, including IL-8, VEGF, and TGFβ-1 [98]. The existence of many types of miRNAs such as miR210, miR126, miR132, and miR21 makes Exo eligible to play important roles in the promotion of angiogenesis [99]. A study on the ischemia mice model has shown that iPSC-derived MSCs Exo can increase microvascular density by stimulating the expression of genes and proteins like PGF, HIF-1α, TGFβ1, VEGFA, and B, angiogenin, bFGF, KDR, and bFGFR [100]. Human ADMSCs-Exo increase the length, number, and branches of microvessels via transferring miR-125a and miRNA-181b-5p/TRPM7 axis [101]. Overexpression of the TRPM7 reverses the effects of miRNA-181b-5p-exosomes on migration and BMECs tube formation. Besides, miR-125a suppresses the angiogenic inhibitor delta-like 4 (DLL4) by targeting its 3′ untranslated region. The increase of endothelial tip cells (CD34^+^ cells) is another mechanism by which miR-125a promotes angiogenesis [102]. Exo loaded with miRNA-181b-5p can upregulate protein expression of HIF-1α and VEGF and can downregulate tissue inhibitor of metalloproteinase 3 [101]. Xin et al. injected MSCs Exo via a tail vein 24 h after MCAO in a rat model. They found an increased vWF positive cells, and neurovascular remodeling, leading to functional recovery 28 days after stroke [29]. Intravenous administration of MSCs Exo enriched with miR-210 led to integrin β3, VEGF, and CD34 expression [103]. It is thought that miRNA-210 is the main hypoxia-induced miRNA through the SOCS1-STAT3-VEGF-C signaling pathway [104]. Exo are enough eligible to transfer and deliver the loaded miRNA-210 through the BBB [105]. This strategy is effective in ischemia/reperfusion models to promote angiogenesis after MCAO [106].

## Exo and neurogenesis after stroke

The process of neurogenesis occurs in both fetus and adulthood. Generally, it includes the proliferation of NSCs, migration of immature neurons and neuroblasts and differentiation into adult neurons, and extension of neurites. This process results in the formation and stabilization of synapses [107]. Neurogenesis plays a crucial role in brain repair and post-stroke recovery [108]. Cerebral ischemia causes endogenous NSCs in the SVZ and SGZ and further migration to the granule cell layer of the DG in the hippocampus. After a stroke, newborn neurons migrate to the infarct zone, replace dead neurons, and incorporate into synaptic circuits, by releasing neuroprotective cytokines [109–111]. However, the physiological potential for neurogenesis after cerebral ischemia is insufficient to repair ischemic brain injury. Of note, the survival rate of newborn neurons is low due to low levels of neurotrophic factors and inflammation caused by ischemia [108, 112, 113]. Therefore, finding new approaches with the potential to enhance neurogenesis can be an effective therapeutic strategy for brain damage improvement and ameliorating functional recovery after stroke [114]. As above-mentioned, Exo can mediate cellular communication through the delivery of their components, including proteins and miRNAs by transmitting genetic information to recipient cells. Among different genetic materials, let-7, miR-124, and miR-9 play a key role in neurogenesis and are highly conserved throughout evolution. These miRNAs are transported to recipient cells via Exo, and trigger the differentiation of NSCs into neurons [115–117]. Following ischemia, the tissue responds to damage by altering the expression of genes such as miRNAs and proteins [118]. miRNAs are proposed as new candidates for gene therapy in neurogenesis. microRNA-124 is widely expressed in the CNS. Decreased miRNA-124 expression in NSCs of SVZ reduces neurogenesis while its activity leads to neural identity acquisition and increased neurogenesis [119, 120]. The expression of miR-124 is elevated in the penumbra zone after ischemic conditions. The introduction of exogenous miR-124 by using agomir or liposomated mimic can help neurogenesis [121]. In a recent study, systemic administration of Exo coated with RVG and miR-124 enhanced the differentiation of NSCs into neurons [21]. The microfluidics technique is a powerful modality that allows the study and observation of the behavior of Exo at the level of secretion, migration, and uptake by neighboring cells. Tracking of Exo containing miR-193a with microfluidics showed that miR-193a facilitates neurogenesis by inhibiting proliferation in undifferentiated sensory neurons (F11 cells). The transfer of Exo from differentiated to undifferentiated neighboring cells leads to stimulation of differentiation in recipient cells. Inhibition of Exo transfer via manumycin-A and treatment of anti-miR-193a in differentiated donor cells blunt neuronal differentiation of undifferentiated recipient cells [117]. Systemic administration of MSCs noticeably enhanced the number of newly formed doublecortin and vWF cells Exo in the MCAO rat model [29]. In the adult brain, neurons in SVZ can communicate with neighboring cells via blood vessels, and CSF [122]. Exo isolated from embryonic CSF of rats and humans contain protein and miRNA components of the IGF signaling pathway. Incubation of embryonic NSCs with CSF Exo induced the IGF/mTORC1 pathway and enhanced the proliferation of these cells [123]. When SVZ-derived NSCs are exposed to proinflammatory cytokines, the release of Exo can stimulate the IFN-γ signaling pathway. The attachment of IFN-γ to IFNGR1 activates the STAT1 pathway in recipient cells. This pathway can regulate EV-associated IFN-γ/IFNGR1complexes, which grafted stem cells probably uses to communicate with the host immune system [124]. Due to the effect of stroke on inducing innate and adaptive immune responses [125], NSC-derived Exo probably play a role in inducing immune responses after stroke.

## Exo and neural plasticity after stroke

Adapting to a changing environment is the mainstay of experiential learning, and neuroplasticity is the basis of learning [126]. During a stroke, the connection between the damaged neurons and the surrounding neurons is lost, and neuroplasticity depending on the location and severity of the lesion, will be destroyed. After a stroke, spontaneous recovery improvement occurs somewhat in humans and animals. This relative improvement occurs in three stages: 1. cell genesis activation and repair, 2. Changing the characteristics of neural pathways and 3. Neuroanatomical plasticity causes the formation of new neuronal connections. Steps 2 and 3 are useful in improving learning after stroke-induced CNS injury [127].

Exo secreted by neurons and glial cells actively coordinate axon growth and myelination [128, 129]. Exo secreted by cultured cortical neurons contain neuronal-specific protein L1 cell adhesion molecule (L1CAM) and the GluR2/3 subunits [130, 131]. Increasing intracellular calcium and depolarization can enhance the release of Exo [130–132]. Exo secreted by neurons contain alpha-amino-3-hydroxy-5-methyl-4-isoxazole propionic acid (AMPA) receptors, and Exo derived from neurites depolarized neurons are enriched with microtubule-associated protein 1b (MAP1b) and miRNAs that are involved in the plasticity of neurites [131, 132]. AMPA receptors and MAP1b are major modulators for the plasticity of synapses and dendrites and enhance axon germination [131–133]. Activation of AMPA receptors via Inducing Local Brain-Derived neurotrophic factor signaling mediates post-stroke motor recovery [134]. Exo derived from cortical neurons increase excitatory amino acid transporter GLT-1 expression in astrocytes by transmitting miR-124 to them and GLT-1 receptor activation modulates synaptic activity by regulating extracellular glutamate [135]. Neurons treated with RARβ2 agonist secrete Exo containing the protein PTEN. These Exo, by transporting PTEN into astrocytes, inhibit their proliferation[136], and preventing glial scar formation by modulation of the PTEN/mTOR pathway, cause axonal sprouting, promoted neurite outgrowth and axon regeneration in adult CNS after spinal cord injury and stroke [137–139].

## Exo and myelination after stroke

Stroke-induced hypoxia causes inflammation and cell death, in oligodendrocytes which are responsible for the production of myelin [140]. White matter lesions within the brain parenchyma (axons and dendrites) after stroke have created a strong incentive to search for effective therapy [141]. In recent years, Exo are the communication transports between neurons and glial cells and have an innate ability to produce myelin sheaths with a suitable three-dimensional structure. Oligodendrocytes secrete Exo in response to neural signals into the extracellular space in a calcium-dependent manner [142, 143]. Exo released from oligodendrocytes are significantly rich in myelin proteins, such as PLP, MBP, MOG, and CNPase, myelin-specific lipids, and other proteins such as 14-3-3 proteins, heat shock proteins, dihydropyrimidinase-related proteins, and peroxiredoxin. These compounds are likely to support axonal traffic due to their role in oxidative stress modulation [143–145]. It seems neurons control myelin biogenesis by regulating the release of oligodendroglial Exo [146]. For example, neuronal glutamate can trigger the release of Exo from oligodendrocytes during the entry of calcium and by acting on the NMDA and AMPA receptors in glial cells. The use of microfluidic chambers has shown the uptake of oligodendrocyte-derived Exo by neurons through the endocytosis, leading to the integrity of axons and somatodendrites under different stresses [146]. There is evidence that Schwann cells participate in PNS myelination via releasing Exo with different cargo compared to oligodendroglial Exo [147]. This issue can explain the higher remyelination efficiency in PNS than in CNS [142]. Schwann cell-derived Exo contain proteins involved in axon regeneration, including carboxypeptidase E (CPE), fatty acid-binding protein (FABP5), fibronectin, flotillin-2, major vault protein (MVP), monocarboxylate transporter 1 (MCT1), neuropilin-2 (NRP2), septin-7 (SEPT7), protein disulfide-isomerase A3 (PDIA3), and syntenin-1 [148]. Exo secreted by myelinating cells like oligodendrocytes and Schwann cells perform an effective role in preserving the myelin and remyelination process by the following mechanisms; (I) Storage and the release of myelin components mediated by neuronal signals, (II) Release of survival factors under physiological and pathological conditions to surrounding axons to maintain axonal myelin, (III) and preservation of the myelin membrane [142]. In many demyelinating diseases and activation of healing processes, resident cells can change exosomal cargo. Serum and CSF exosomal factors such as MBP, MOG, PLP, SMase, and Let-7i can be considered diagnostic factors for pathological conditions (Tables 1, 2) [142]. For example, in SCI, the presence of retinoic acid in Exo is an essential diagnostic factor in increasing neurite outgrowth and regeneration [149]. Many factor such as GDNF, FGF-1, miR-199a-5p, miR-145, miR-134, BDNF, IGF-1, and NGF have been found in MSCs Exo that have potential therapeutic potential for demyelinating diseases [142].

**Table 1:** The role of Exo in the diagnosis, therapy, and improvement of stroke-induced injuries in animal models

Exosome	miRNA	The role	Mechanism	Reference
MSCs Exo	–	Enhancing functional recovery, plasticity, neurite remodeling, neurogenesis, and angiogenesis. in the neurovascular system in the MCAo rat model	Increasing the density of axons and synaptophysin in the ischemic boundary zone of the cortex and striatum	[29]
rabies virus glycoprotein (RVG)-decorated exosomes (RVG-Exo)	HMGB1-siRNA	Therapeutic effect for ischemic stroke in the MCAo model	Reducing TNF-α, apoptosis, and infarct size in the brain	[153]
MSCs derived Exo	miRNA-17-92	Promoting neurological recovery after stroke in the neurovascular system in the MCAo rat model	Enhancing oligodendrogenesis, neurogenesis, neurite remodeling/neuronal dendrite plasticity and axonal outgrowth in the ischemic boundary zone (IBZ) by targeting phosphatase and tensin homolog to activate the PI3K/protein kinase B/mechanistic target of rapamycin/glycogen synthase kinase 3β pathway	[30]
M2 microglia-derived Exo	miRNA-124	Reduction of ischemic brain injury and behavioral impairments three days after transient brain ischemia in the MCAo mouse model	Reducing neuronal apoptosis, and infarct volume, and increasing survival of neurons by targeting ubiquitin-specific protease 14 (USP14)	[154]
ADSCs Exo	miRNA-30d-5p	The protective effect and cerebral injury prevention after acute ischemic stroke in the rat model of AIS and an in vitro model of oxygen- and glucose-deprived (OGD)	Suppression of autophagy-mediated microglial polarization to M1	[86]
lipopolysaccharide-stimulated macrophage RAW264.7 cell line (LPS-Exo)	–	Neuroprotection effect and functional recovery after ischemic stroke in a rat model of transient focal cerebral ischemia	Reduction of brain infarct size via polarization from the M1 phenotype to the M2 phenotype and suppression of inflammation	[51]
Endothelial progenitor cell-derived Exo	miRNA-126	Functional recovery improvement in the C57BL/6 mice that received moderate treadmill exercise (10 m/min) for 4-wks and then were under MCAO surgery	Reduction of infarct volume, apoptosis, and promoting microvessel density, angiogenesis/neurogenesis, and better sensorimotor functions by targeting BDNF, p-TrkB/TrkB, and p-Akt/Akt signaling pathway	[155]
Adipose-derived mesenchymal stem cells-derived Exo	–	Neurological function improvement after acute ischemic stroke in the MCAo rat model	Decreasing ischemic volume, inflammation, and oxidative stress and increasing angiogenesis and return of blood to the ischemic area	[77]
Bone marrow-derived mesenchymal stem cells (BMSCs)-derived Exo	microRNA-138-5p	Neuroprotection to astrocytes and reduction of neurological impairment following ischemic stroke in the MCAO mice model	Prevention of astrocytes apoptosis and reduction of inflammatory factors expression through down-regulating neutrophil gelatinase-associated lipocalin (LCN2)	[156]
Exo with rabies virus glycoprotein (RVG)	miRNA-124	Enhancing cortical neural progenitors to obtain neuronal identity and protection against ischemic injury	Promoting neurogenesis, angiogenesis, and neural plasticity	[21]
ADSCs Exo	miRNA-126	Functional recovery improvement after stroke in the MCAo rat model	Enhancing von Willebrand factor (an endothelial cell marker) and doublecortin (a neuroblasts marker) expression, increasing cell proliferation and neurogenesis, inhibiting microglial activation, inflammatory factors expression, and decrease of neural death	[157]
Exosomal serum miRNA126	miRNA-126	Distinguishing severe permanent ischemia from milder injury after transient ischemia through changes in exosomal serum miRNA-126 in the transient focal cerebral ischemia rat model	Reducing of serum miR-126 at 3 h after permanent ischemia but not transient ischemia and the serum miR-126 levels back the closing to baseline in both permanent and transient ischemia after 24 h	[158]
Bone marrow stem cells Exo	–	Neuroprotection and functional recovery in the MCAo rat model	Reduction of infarct volume, diminishing of GFAP positive cells and lipid peroxidation, and downregulating of NLRP1 and NLRP3 genes represent a lower rate of cell death	[159]
Exo were extracted from the peri-ischemic striatum	miRNA 146b	Neurological injury improvement after ischemic stroke in the MCAo rat model	Increasing exosomal biomarkers TSG101 and CD81 and enhancing differentiation of neural stem cells into neurons in the peri-ischemic striatum	[160]
Human neural stem cells derived Exo	hsa-miRNA-206, hsa-miRNA-133a-3p and hsa-miRNA-3656	Therapeutic ability and improvement of behavioral and structural outcomes in ischemic stroke in the MCAo rat model	Promoting cell proliferation and cell survival and reducing cell apoptosis in vitro by stimulating interferon-gamma (IFN-γ)	[161]
CSF and plasma-derived Exo	miRNA-122-5p and miR-300-3p	Potential blood-based biomarkers for the transient ischemic attack in the MCAo rat model	Downregulating of plasma exosomal rno-miR-122-5p in 10 min ischaemic rats and upregulating of plasma exosomal rno-miR-300-3p in 5 min ischaemic rats	[162]
MSCs derived Exo	miRNA 133b	Functional recovery after stroke in the MCAo rat model	Neurite remodeling/brain plasticity in the ischemic boundary zine with a contribution from a stimulated secondary secretion of neurite-enhancing exosomes from astrocytes	[31]
Bone marrow-derived MSCs Exo	miRNA-210	Enhancing angiogenesis, brain tissue repair, and animal survival rat after cerebral ischemia in the transient MCAo mouse model	Increasing integrin β3, vascular endothelial growth factor (VEGF) expression, and upregulating CD34	[103]
Enkephalin delivery using Exo	–	Improving neurological recovery and neurological score after stroke in the transient MCAo rat model	Suppressing neuron apoptosis caused by glutamate, increasing neuron density, and reducing the levels of LDH, p53, caspase-3, and NO	[163]
Exo secreted from human umbilical cord blood (HUCB)-derived MSCs under in vitro hypoxic conditions	–	Decreasing the post-stroke brain injury and ameliorating the neurological outcome in the transient MCAo rat model	Diminishing the infarct volume and swelling of the ipsilateral hemisphere	[164]
Astrocyte Exo	miRNA-7670-3p	Improving ischemic brain damage in the MCAo mouse model	Upregulating of shuttled circSHOC2astrocyte-derived exosomes, reduction of neuronal apoptosis by regulating neuronal autophagy via the miR-7670-3p/SIRT1 Axis	[89]
MSCs Exo	–	Restoring white matter integrity and promoting neurovascular remodeling and functional recovery in adult male rats were subjected to intracerebral hemorrhage (ICH)	Improving lesion size, fiber tract integrity, axonal sprouting, and white matter repair markers	[141]
Human umbilical cord mesenchymal stem cells (HUC-MSCs) Exo	–	Improvement of cognition and promoting oligodendrogenesis and remyelination after stroke in the transient MCAo rat model	Overexpression of chemokine receptor type 2 (CCR2) in the HUC-MSCs-derived exosomes, inhibiting activation and migration of CCL2-induced hematogenous macrophage and increasing and microglia/macrophage M2 polarization	[165]
Bone mesenchymal stem cell Exo	miRNA-29b-3p	Improving ischemic brain injury, suppressing apoptosis, and enhancing angiogenesis in the MCAo rat model	PTEN negatively regulation and Akt signaling pathway activation	[166]
Endothelial progenitor cells (EPCs) Exo	miRNA‐126	Decreasing acute damage and improving neurological function recovery after ischemic stroke in the MCAo mouse model	Downregulating cleaved caspase‐3, upregulating vascular endothelial growth factor receptor 2 (VEGFR2), diminishing infarct volume. enhancing cerebral blood flow (CBF) and cerebral microvascular density (MVD) ameliorating angiogenesis and neurogenesis	[167]
Ischemic cerebral endothelial cell-derived Exo	–	Maintaining vascular and neuronal homeostasis in the normal and ischemic brain in the transient MCAo rat model	Enhancing axonal growth and axonal remodeling in cortical neurons	[168]
Bone marrow mesenchymal stem cells Exo harvested from type two diabetes rats	–	Ameliorating of neurological function and restoration of neurons after stroke in type two diabetes rats in the transient MCAo rat model	Decreasing post-stroke weight loss, enhancing tight junction protein ZO-1, promoting the integrity of the blood–brain barrier (BBB), remodeling of the white matter, the density of axon and myelin, oligodendrocytes and oligodendrocyte progenitor cell numbers, primary cortical neuronal axonal outgrowth, expression of miR-9 target ABCA1 (ATP-binding cassette transporter 1) and IGFR1 (Insulin-like growth factor 1 receptor) and diminishing activated microglia, M1 macrophages, inflammatory factors MMP-9 (matrix metalloproteinase-9), MCP-1 (monocyte chemoattractant protein-1) in the ischemic border area of the brain and decreasing of miR-9 expression in serum	[169]

**Table 2:** Diagnostic effect of exosomes in stroke patients

Exosome	miRNA	The role	Mechanism	Reference
Exo in the serum of acute ischemic stroke patients 24 h after stroke	miRNA-134	Diagnosis and prognosis of stroke	Enhancing of serum interleukin 6 (IL-6) and plasma high-sensitivity C relative protein (hs-CRP) expression	[170]
Exo in human circulating blood with acute ischemic stroke patients 72 h after stroke	miRNA-223	Diagnosis and prognosis of stroke	Correlation with acute ischemic stroke occurrence, stroke severity, and short-term outcomes	[171]
Exo in the serum of acute ischemic stroke patients	miRNA-9 and miRNA-124	Diagnosis and prognosis of stroke	A positive correlation between both miR-9 and miR-124 levels with National Institutes of Health Stroke Scale (NIHSS) scores, infarct size, and concentrations of IL-6 in the serum	[172]
The plasma-derived Exo in patients with different phases of ischemic stroke	miRNA-21-5p	A biomarker to Diagnosis and identifies in early-stage of ischemic stroke	The plasma exosomal miR-21-5p levels are significantly higher in patients than in the control group	[173]
The plasma-derived Exo in patients with acute phase and subacute phase of ischemic stroke	miR-422a and miR-125b-2-3p	A blood-based reference for monitoring and diagnosing patients with ischemic stroke	Decreasing plasma exosomal miRNA-422a and miR-125b-2-3p expression in the subacute phase group and increasing miRNA-422a levels in the acute phase group rather than in the control group and reduction of plasma exosomal miR-422a and miR-125b-2-3p expression in the subacute phase group than in the acute phase group	[174]

## Clinical relevance and prospects

According to current data, there is a few studies that applied Exo in the treatment of brain stroke. By June 2022, the public clinical trial database https://clinicaltrials.gov presented about three clinical trials in patients with stroke. In one study, the reparative properties of allogenic MSC-derived Exo are investigated in patients with acute ischemic stroke. Another study will focus on the diagnostic value of blood Exo cargo in stroke patients after rehabilitation. In the last study, the role of acupuncture-induced Exo will be investigated in the treatment of post-stroke dementia. Along with these data, IFN-γ-primed human NSCs produce Exo with the ability to promote cell proliferation and decrease apoptosis in ischemic rats [150]. In another experiment, human urine stem cells Exo accelerated neurogenesis in an experimental stroke rat model by the regulation of the miR-26a/HDAC6 axis [151]. Furthermore, Exo from human cardiosphere-derived cells can improve recovery in the rabbit embolic stroke model after reperfusion [152]. These data suggest that Exo can be used as a novel promising strategy for brain ischemia, highlighting their eligibility in the clinical setting.

## Conclusion

Numerous emerging studies show the potential of Exo in treating a variety of pathologies after ischemic conditions. Naive and engineered Exo can be effective in the control of inflammation, apoptosis, autophagy, neurovascular remodeling, angiogenesis, neurogenesis, synaptic plasticity, and myelination after stroke. Along with the regenerative potential of Exo, these nano-sized particles can cross the BBB prepared from an allogenic source with low immunogenicity, and a low risk of developing a tumor and occlusion in the arteries raised new hopes for the treatment of stroke patients.

## Data Availability

Not applicable.

## Abbreviations

BBB: Blood–brain barrier
CREB: CAMP response element-binding protein
CNS: Central nervous system
DG: Dentate gyrus
ESCRT: Endosomal sorting complexes required for transport
Exo: Exosomes
ECM: Extracellular matrix
ERK1/2: Extracellular signal-regulated protein kinase 1/2
EVs: Extracellular vesicles
HSPs: Heat shock proteins
HRS: Hepatocyte growth factor‐regulated tyrosine kinase substrate
MHC-II: Histocompatibility complex class II
iNOS: Inducible nitric oxide synthase
IL: Interleukin
ICH: Intracerebral hemorrhage
ILV: Intraluminal vesicles
I/R: Ischemia-reperfusion
MSC: Mesenchymal stem cell
MCAO: Middle cerebral artery occlusion
MVB: Multivesicular bodies
NSCs: Neural stem cells
NMDA: *N*-Methyl-d-aspartate
NF-κB: Nuclear factor-kappa B
OGD: Oxygen‐glucose deprivation
PtdIns (3)-P: Phosphatidylinositol 3-phosphate
PEDF: Pigment epithelium-derived factor
RVG: Rabies virus glycoprotein
RARβ2: Retinoic acid receptor β2
SIRT1: Sirtuin 1
SGZ: Subgranular zone
SVZ: Subventricular zone
tPA: Tissue plasminogen activator
TRPM7: Transient receptor potential melastatin 7
TNF-α: Tumor necrosis factor-alpha
VPS4: Vacuolar protein‐sorting‐associated protein 4
AMPA: α-Amino-3-hydroxy-5-methyl-4-isoxazole propionic acid
